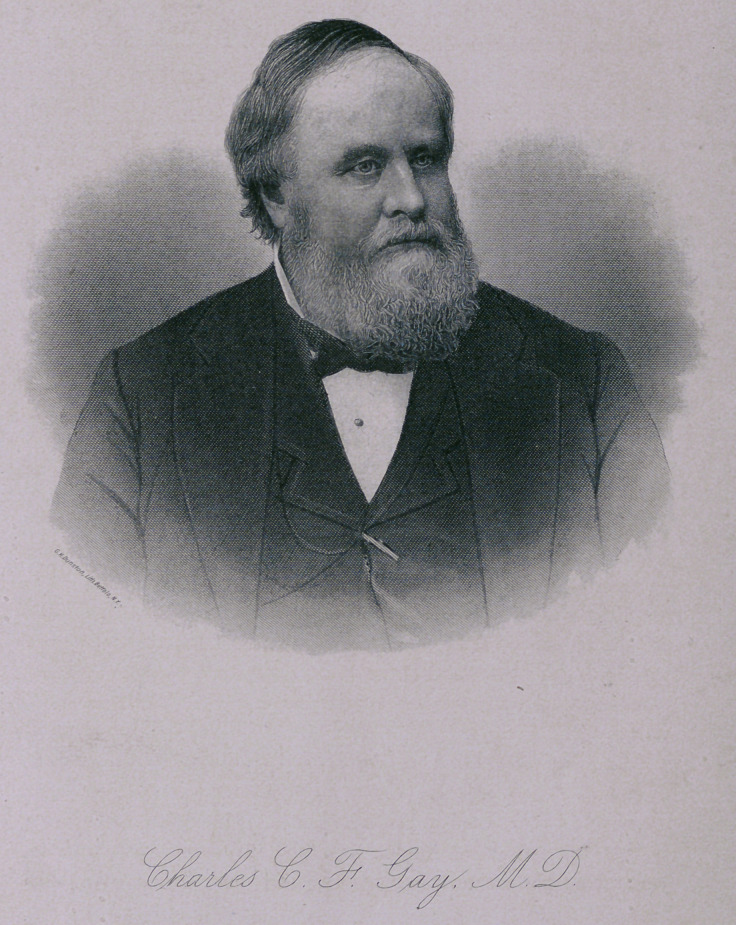# Charles Curtis Fenn Gay, M. D.

**Published:** 1886-05

**Authors:** 


### Editorial.

### Charles Curtis Fenn Gay, M. D.

Once more we are called upon to mourn the loss of a distinguished member of our profession, and the physicians of Buffalo, without exception, are deeply grieved at the death of one who was so dearly beloved. Kindness and Christian charity were the marked characteristics of Dr. Gay, and through all his dealings with his fellow-practitioners and patients, these shone as a bright light illuminating his path. In the defense of right, the doctor has struck many a powerful blow. Not a few unscrupulous persons in this city can testify to the weight of that strong right arm of his when wielded to defend the right and to uphold a true principle. His charity and power of forgiving were not less than his strength in the defense of right. His death is a great loss to this community. He will be missed in the medical society and in the lecture hall, where he was listened to with attention and profit; in the social circle, where his genial and pleasant face was always welcome; in the sick room, where his dignified bearing, wise counsel and abundant sympathy were of themselves restorative; in the church, of which he was a consistent member, and where he was always ready to proclaim that faith in the supreme and overruling Providence, but, most of all, he will be missed in the family circle, for he was a kind and loving husband and father. To his most estimable wife and companion of so many years, our hearts go out in deepest sympathy.

Dr. Gay was born in Pittsfield, Berkshire County, Mass., January 7, 1821, and was the son of William Gay, Jr., who was a native of Worcester, in the same State. The common ancestor of the Gay family in America was John Gay, who, with his wife, came from the western part of England, making the voyage in

Charles C. F. Gay, M.D.

the ship "Mary and John," and landing in this country on the 30th of May, 1630. He first settled at Watertown, Mass., but a few years afterward removed to Dedham, where, in the year 1688, he died, having attained to a ripe old age. Dr. Gay is a lineal descendant in the seventh generation from the founder of the family.

Among the prominent members of the Gay family may be mentioned three doctors of divinity. The late Edwin M. Stanton, secretary of war during the administration of President Lincoln, was a distant cousin of the mother of Dr. Gay, who is now living at the age of 91, vigorous both in mind and body. While he was still a boy, Dr. Gay's parents removed to Lebanon Springs, Columbia County, N. Y. He received his preliminary education in the select schools of that neighborhood, one of which was the classical school of Prof. John Hunter, of New Lebanon. At a later period (1843) he attended the collegiate institute at Brockport, Monroe County, N. Y. He commenced the study of medicine in 1844, entering the office of Dr. Joseph Bates, of Lebanon Springs. Soon afterward he went to Pittsfield, Mass., where he studied under Dr. H. H. Childs, who, in 1843, had been Lieutenant-Governor of the State. He also attended a course of instruction in the Berkshire Medical College, and one in the medical school at Woodstock, Vt. A third course was attended at the former institution, from which, in the fall of 1846, he received his medical degree. The great center of medical instruction at that period was Philadelphia, whither, after graduation, Dr. Gay repaired, and completed his studies by attending the winter course of lectures in the Jefferson Medical College. In 1847, he commenced practice at Bennington, Vt., removing in a few years to Byron, Genesee County, N. Y. After practicing in this place some four or five years, he removed to Buffalo, where he has since resided.

In 1855, at the organization of the Buffalo General Hospital, he was chosen consulting surgeon, and three years subsequently was appointed attending surgeon, a position which he held till 1884.

Among Dr. Gay's papers are interesting memoranda of the first efforts to organize the Buffalo General Hospital. Meetings were held at his office, being attended by many doctors and business men. Among the doctors mentioned frequently are Drs. Strong and Wilcox. Through him, his father-in-law, the late George W. Tifft, and other friends were interested in this movement. Buffalo owes to no one more than to Dr. Gay in the foundation of this great public charity.

In 1861, he was appointed, by the Union Defense Committee of Buffalo, surgeon-in-charge of Fort Porter, and, while at this post, examined and had charge of the Forty-ninth Regiment, N. Y. V., Col. Daniel D. Bidwell commanding. He is one of the founders of the Society of Natural Sciences of Buffalo, and served on the original board of directors. He was also curator of botany in the institution at an early period in its history. On the organization of the Buffalo Surgical Infirmary, in 1876, he was chosen surgeon-in-chief. Always an advocate of higher medical education, he was appointed by the authorities of Niagara University upon the establishment of the medical department to the chair of clinical surgery, and held that position until ill health compelled his resignation, when he was appointed emeritus professor.

He was married, in January, 1854, in Buffalo, to Sarah A., daughter of the late George W. Tifft, Esq., an old and respected resident of this city. Dr. Gay's life has been devoted to the science of medicine, and he held a high rank in the profession, both as physician and surgeon. He has been a permanent member of the State Medical Society since 1861. He was also a member of the Erie County Medical Society, and has been a president of that body. On a number of occasions, he has been delegate to the conventions of the American Medical Association, and has made verbal reports on surgical operations before that distinguished body. He was also delegate to the British Medical Society in 1885.

His reports and contributions to medical literature have been numerous. Among them the following are specially entitled to mention: "Erysipelas; its Constitutional Origin and Treatment," 1859; "Medical Progress," 1862; "Hints Regarding the Management of Fractured Bones," 1867; "Placenta Previa," 1868; "Uterine Surgery," 1868; "Uterine Displacements, and their Surgical Treatment," 1868; "Vesico-Vaginal Fistula," 1868; "Unavoidable Hemorrhage," 1869; "Puerperal Eclampsia," 1869; "Two Cases of Labor Complicated by Presence of Uterine Tumors," 1869; "Fracture of Acetabulum," 1884; "Protoxide of Azeote as an Anæsthetic Agent," (translated from the French); "Intestinal Invagination, etc.," "Retroversion of the Impregnated Uterus and Spontaneous Reposition," "Encephaloid Tumor, etc.," "Hernia," "On Retention of Urine from Traumatic Stricture," "Varicose Veins," "Radical Cure of Hydrocele," "Operation for Procedentia Uteri," "Aneurismal Tumor Following Penetrating Wound of the Thorax," "Radical Cure of Inguinal Hernia," "Case of Ligation of the Left Sub-Clavian Artery."

Of late years he has devoted his attention more especially to surgery, and was engaged in preparing a work on that branch of medical science. For over a year the doctor has been ailing, owing to a sickness contracted while in the discharge of his duty at the General Hospital. For several months he was quite ill at home. Being advised to take a trip to Europe, he did so, returning in the fall comparatively well. On his return, he was given a reception and banquet at the Genesee House by the faculty of the Niagara Medical School. The doctor then seemed fully restored to health, but it was not long before he became again seriously ill. He and his physicians realized, from the state of affairs at the outset, that he could not recover. After a painful illness of several weeks, he died March 27, 1886. He maintained throughout a cheerful mood, and frequently proclaimed his faith in God and his adherence to the Christian principles which had been his guide for many years.

## Figures and Tables

**Figure f1:**